# Book Reviews

**Published:** 1832-09

**Authors:** 


### CRITICAL ANALYSES.

Quæ laudanda forent, et quæ culpanda, vicissim,
Illa prius, cretâ; mox hæc, carbone, *notamus*.—Persius.

*On the Sources and Nature of the Powers on which the Circulation of the Blood depends.* By A. P. W. Philip, M.D., F.R.S. L. & E. (*Philosophical Transactions* for 1832.)

In a recent number of this Journal we noticed, at some length, Dr. Marshall Hall's work upon the Circulation. We had taken more than usual interest in the subject at that moment, from having recently had an opportunity of seeing the phenomena described by Dr. Hall with our own eyes: we had traced the minute vessels, both arteries and veins, and had been highly interested by the distinction (first *fully* made at that time,) between them and the *true* capillaries. We had also been privy to some proceedings at the Royal Society, which we did not think calculated to add to the credit of the parties concerned. We object, firmly object, to the occasion on which Dr. Philip brought forward his paper. The only way in which the affair could be justified, would be the proof that Dr. Philip's paper alone was the correct one. Whether this be so or not, our readers will judge when they have gone through our brief remarks upon it.

We shall take Dr. Philip's statements in the order in which they are given by him.

We find Dr. Philip first disputing, amongst some other opinions, that of Sir David Barry upon the influence of the acts of inspiration on the venous circulation. Dr. Philip states,

"It has been supposed that what has been called the resilience of the lungs, that is, their tendency to collapse, by relieving the external surface of the heart from some part of the pressure of the atmosphere, is a principal means of causing it to be distended with blood, the whole weight of the atmosphere acting on its internal surface through the medium of the blood which is thus propelled from the veins into its cavities; and in this way it has been supposed that the motion of the blood through the whole of the venous part of the circulation is maintained. A similar effect has been ascribed to the act of inspiration, which it is evident must operate on the same principle; and this opinion has even been sanctioned by the Report of a Committee of the Royal Academy of Sciences of Paris,* and in this country by men whose authority is deservedly high; and the effect of these causes, it is asserted, is increased by the elastic power of the heart itself."

In order to controvert this opinion of Sir David Barry, Dr. Philip adduces the following experiment:

* Report on Dr. Barry s Paper, by Baron Cuvier and Professor Dumeril.

"*Exp.* A rabbit was killed in the usual way by a blow on the occiput, and the chest opened on both sides so as freely to admit the air. The lungs were then inflated eight or ten times in the minute by means of a pipe introduced into the trachea; the circulation was found to be vigorous. On laying bare one of the femoral arteries, it was observed to pulsate strongly; and on wounding it, the blood, of a florid colour, indicating that it had undergone the proper change in its circulation through the lungs, gushed out with great force; and on introducing the hand into the thorax, the heart was found to be alternately distended and contracted as in the healthy circulation."

We do not propose to defend any of the opinions which Dr. Philip has undertaken to controvert; not even that of Dr. Barry; but we do think it right to point out the kind of reasoning employed by Dr. Philip. That gentleman observes,

"It appears from these experiments that the circulation was vigorous when none of the causes to which the motion of the blood in the veins have been ascribed existed. In the first experiment the chest being freely opened on both sides, so that the play of the lungs on inflating them could be seen, all effect on the heart either of the resilience of the lungs or the act of inspiration, was evidently prevented: and in the second, it was proved that no sensible elasticity of the heart existed; yet while artificial respiration was performed we could perceive no abatement in the vigour of the circulation."

Now this reasoning amounts, in fact, to this: A carriage is said to be drawn by three pairs of horses; the leaders are removed, and "no abatement" in the speed of the carriage is perceived: *therefore,* that pair of horses did not assist in drawing the carriage! After the thorax was opened, the heart continued to beat, and to force the blood *through the arteries: therefore,* when the thorax was entire, and the acts of inspiration going on, the circulation could not be promoted by them *through the veins!*

Dr. Philip next attempts to establish an opinion of his own, that the action of the capillary vessels greatly assists the circulation of the blood:

"Here, as in the former instance, it is better to appeal to the evidence of direct facts than to any train of reasoning; and there is no want of such facts to determine the point before us, some of which I formerly had the honour to lay before the Society, and others are stated in my Treatise on the Vital Functions. The most decisive is, that the motion of the blood in the capillaries continues long after the heart has ceased to beat, and the animal, in the common acceptation of the term, is dead, even in the warm-blooded animal, for an hour and a half or two hours, and it is not for some time sensibly affected by the heart's ceasing to beat; nor does this arise from some imperceptible impulse still given by the heart, because when all the vessels attached to this organ are secured by a ligature and the heart cut out, the result is the same.

"That the circulation in the capillary vessels is independent of the heart, may be shewn by various other means. On viewing the motion of the blood in them, with the assistance of the microscope, it may generally be observed that it is moving with different degrees of velocity in the different vessels of the part we are viewing, frequently more than twice as rapidly in some than in others. Were the motion derived from a common source, this could not be the case. It is impossible, in the motion of the blood in the capillaries, in the least degree to perceive the impulse given by the beating of the heart, which causes the blood in the arteries to move more or less per saltum, the motion of the blood in the former being uniform as long as they retain their vigour, and the necessary supply of blood is afforded from the larger vessels. I have found by experiments very frequently repeated, that the motion of the blood may be accelerated or retarded in the capillaries by stimulants or sedatives, acting not through the medium of the heart, but on these vessels themselves."

Here are four distinct assertions: the *first* is, that the capillary circulation continues after the influence of the heart is withdrawn by ligature and excision. Having seen this experiment repeated, we are prepared to say that the assertion is incorrect. We can further explain Dr. Philip's error, being satisfied that it has arisen out of various causes of motion in the blood of the capillary vessels, external to themselves, and chiefly the varied degrees of extension or stretching of the membranes in which they are inclosed. If these sources of error be avoided, the circulation ceases in the true capillaries, a few moments after the removal of the influence of the heart.

The *second* proposition is, that the blood moves with different degrees of velocity in the different vessels (the true capillaries, of course,) in the part viewed. Dr. Hall employed an infinitely better microscope than Dr. Philip; and we can assert that this statement is also erroneous. If unequal extension of the membrane examined, and similar influential causes, be avoided, the flow of the blood is precisely the same, and uniform in all the capillary vessels of the part.

Dr. Philip asserts, in the *third* place, that "it is impossible, in the motion of the blood in the capillaries, in the least degree to perceive the impulse given by the beating of the heart." We, on the other hand, venture to assert that nothing is so possible, nothing so easy, nothing so common. Dr. Hastings says the same thing; so does Dr. Thomson. The former of these authors says distinctly, "The conclusions to be drawn from these experiments appear interesting. From the first we learn, contrary to what is maintained by Bichat and some other physiologists, that *the action of the heart influences the circulation in the capillaries, and that it even extends to the veins:* for in what other way can we account for the sensible impulse given to the venous blood at each systole of the ventricle, but by supposing that the impetus given to the mass of blood was felt in these tubes."* It is only necessary, indeed, to impede the flow of blood along the thigh of the frog, by a ligature lightly applied to it, to be enabled to count the beat of the heart, alike in arteries, capillaries, and veins.

The *fourth* assertion of Dr. Philip is, that the motion of the blood may be influenced, in the capillaries, by the application of stimulants or sedatives to these vessels themselves. So may birds be caught by putting salt upon their tails. But, first, should not Dr. Philip have established that there *are* such things as *capillary* VESSELS, as distinguished from mere CANALS? This would have been philosophical. We can only say, that we have sought for such proof in vain. MECKEL, WEDEMEYER, DOLLINGES, and many others, state positively that there are no such vessels! Then how has Dr. Philip applied stimulants and sedatives to them? As well might a philosopher speak of having placed his finger upon the poles of the earth. Dr. Philip's experiments were made, in fact, upon *membranes inclosing canals merely*, as far as our present knowledge extends, and not upon the "capillary *vessels* themselves." And if all this be really true, what becomes of Dr. Philip's experiments upon the influence exerted by the brain upon the capillaries, and his boasted theory of inflammation? We greatly fear Dr. Philip is not *au niveau*, as we say, in his anatomy; and that he has, in many of these experiments, excluded the influence of other agents from the real functions of the true capillaries. In that of stimulants or sedatives applied, as is said, to the capillaries, the influence of their effect on the membranes is not excluded; and, in that of crushing or irritating the brain, the influence of the heart itself, of the arteries, and of the muscular system, is not removed. We must have simpler, clearer, experiments than these, before any inference of a satisfactory kind can be drawn from them.

Dr. Philip adds,

"If the circulation in the capillaries be thus independent of the heart, it is evident that the influence of that organ cannot extend to the veins. On comparing the whole of the foregoing circumstances, is it not a necessary inference, that the motion of the blood in the veins, like that in the capillaries, depends on the power of these vessels themselves? But that we may not trust to any train of reasoning, where it is possible to have recourse to direct proof, I made the following experiment, with the assistance of Mr. Cutler.

"*Exp.* In the newly dead rabbit, in which the circulation was maintained by artificial respiration, the jugular vein was laid bare for about an inch and a half; a ligature was then passed behind the part of the vessel nearest to the head, and the animal was so placed that the vein was brought into the perpendicular position, the head of the animal being undermost, so that it was necessary for the vein, in conveying the blood to the heart, to convey it perpendicularly

* Hastings on Bronchitis, p. 49.

against its gravity. The ligature, which was placed at what was now the lowest part of the exposed portion of the vein, was suddenly tightened, while Mr. Cutler and myself observed the vessel. The blood in the part of the vein between the ligature and the heart was instantly and completely expelled, as the transparency of the vessel enabled us to perceive. The vessel itself wholly collapsed, proving that all its blood had entered the heart, so that to a superficial view there seemed to be no vessel in the part where a large dark-coloured vein had just before appeared. In the mean time, on the other side of the ligature, the vein had become gorged with blood.

"In the foregoing experiment we see the blood rising rapidly against its gravity, where all causes external to the vessel on which the venous part of the circulation has been supposed to depend had ceased to exist, and the vis à tergo was wholly destroyed by the ligature."

Did philosopher ever perform so insufficient an experiment? Does Dr. Philip really mean to assert that the blood was propelled out of this "wholly collapsed" vessel by "the power of this vessel itself?" Does a vessel which empties itself by its own power *collapse?* This experiment was repeated in the presence of two of the best physiologists and most accurate thinkers of this metropolis. The vein was found to empty itself, or fill itself, just as it was made or allowed to do so by pressing or not pressing on the parts behind it!

We have not time or space for more remarks upon Dr. Philip's paper: we think we have said enough to prove that it was very little worthy of a place in those records of the science of our country in which it appeared. There are seven or eight propositions contained in it, and we verily believe six or seven of them are at variance with the facts as established by the recent progress of physiology, to many of which we ourselves have been witness. We advise Dr. Philip to read the works of those authors to whom we have referred, first dispossessing himself of old and antiquated notions, and then going afresh to experiment, taking greater care that each experiment will be a sufficient experiment; which certainly cannot be said of the two we have quoted in this article.

We are too much interested in this subject not to keep our attention fixed upon it. We are anxious for the truth, and care not for what has been called authority. Give us facts, good and *sufficient* facts, and sound reasoning, and we shall adopt them.

*Two Lectures on the Primary and Secondary Treatment of Burns.* By HENRY EARLE, F.R.S., Surgeon Extraordinary to the King, Surgeon to St. Bartholomew's Hospital, &c.— 8vo. pp. 59, with Plates. Longman and Co., London.

(Concluded from page 151.)

WE have already expressed our favorable opinion of these lectures, in which much practical information, the result of patient inquiry and philosophical acumen, has been condensed into a small space, and communicated in a pleasing style: it, therefore, only remains for us to select those parts from the second lecture in which the author explains the treatment he pursues, and recommends to be pursued, in the secondary stages of severe burns and scalds; and chiefly with regard to what he considers "the best means for those unseemly deformities, contractions, and lamenesses, which so frequently ensue in these cases when the counteracting influence of art is not skilfully interposed."

The cautions as to the mode in which dressings should be applied are important, and the more so that, although commonly given, they are so seldom attended to; and we have known ulcers absolutely kept from healing by the injudicious mode of dressing.

"On examining a healthy healing ulcer, we shall always find at the circumference a marginal line of deep red, which has a smooth polished appearance when closely viewed, differing from the irregular granulating surface. This is the newly deposited skin: and becomes more apparent after an ulcer has been exposed for some minutes. If dry lint, which is often the best and only stimulus wanted for a healthy sore, be applied over this margin, it will adhere so closely to it, that, unless great care be taken to moisten the dressings, in removing them the newly deposited skin will be destroyed, and the surface will bleed, causing considerable pain to the patient; and thus destroying, in one minute, a whole day's reparation. This may be effectually obviated by one of two plans: either by applying narrow strips of lint, smeared with some unctuous substance, round the whole margin of the wound, and filling up the interspace with dry lint, over which a large pledget should be applied, to exclude the air, and prevent the pus which is secreted from encrusting on the wound; or by accurately fitting the size of the lint, so as not to encroach upon the margin of the wound, and then covering the whole with a pledget larger than the entire surface." (P. 33.)

In the dressings themselves we do not perceive any thing more recommended than is commonly employed; nor, indeed, do we think there is much occasion for other means; hence our subsequent extracts will relate to the prevention and removal of the deformities so often the consequence of the injuries in question.

"I come now to speak of the particular local treatment which will be required in cases where the burn has occurred in the flexures of joints, and in the front of the neck; in all situations, indeed, where contractions are likely to ensue. You are, no doubt, aware that, after the healing of a wound, the cicatrix almost invariably will be found to occupy a smaller space than the original ulcer. This depends partly on the retraction of the surrounding healthy skin after the separation of the slough; of which you have a familiar example in making an issue with caustic potash; but it will be found to depend more particularly on the changes which take place during and after the healing of the wound. The process by which these are effected consists in an absorption of the granulations on which the new skin has been deposited, by which the cicatrix is made to occupy a much smaller extent than the originally ulcerated surface. Perhaps it would be speaking more correctly, to say that the granulations, which are at first florid and extremely vascular, after having deposited the new skin, receive a smaller proportion of blood, become paler and diminished in bulk, and consequently occupy much less surface for the new skin. In many cases, such as amputation, where sometimes sufficient integuments have not been saved to cover the bones, this process is very salutary, as it is essential to have the smallest possible extent of new skin on a surface which is to be subject to much pressure; so also the curative operations in ectropeon and entropeon, are adopted on this well-known principle of the contraction of the cicatrix. But in extensive ulcers, occurring in the neighbourhood of the neck, and in the flexure of joints, it often causes the most distressing contractions and deformities. The force with which this gradual process acts is truly astonishing; as the repeated drop of water will, in time, undermine the firmest rock, so will this slow but powerful process effect the most extraordinary changes in the form. I have repeatedly known it draw down the chin upon the sternum; and approximate the shoulders, by the partial absorption of the clavicles, so as to alter the dimensions of the thorax. I have had many instances in which the fore-arm has been permanently bent upon the arm, so as to bring the thumb in contact with the point of the shoulder. In the course of last year, two remarkable instances occurred in this hospital, of which I have an opportunity of presenting you with very faithful drawings. In the one, not only is the whole head bowed down towards the sternum, but actually the firm bone of the lower jaw has been curved downwards, so as only to admit of the last molar teeth coming in contact; the mouth being kept permanently open, and the direction of the incisor teeth being altered so as to cause them to project nearly in a horizontal line. In the other, the arm was pinioned to the side, and the hairy scalp was drawn many inches down the back between the scapulæ. At the present time there is a melancholy instance in the house, where the fingers and thumbs of both hands are drawn together in the palms, so as to render the extremities nearly useless. These examples will suffice to shew the extent and nature of such contractions.

"The important question now arises, is it possible, by any human art, to prevent such occurrences? or does this process baffle all our efforts to control it? In other words, are such consequences as I have now represented to be attributed to the mal-practice and negligence of the surgeon? or are they unavoidable? I have no hesitation in saying, that in a large majority of cases such consequences may be prevented, by the well directed efforts of art, assisted by machinery. It is now nineteen years since I first called the attention of the profession to this subject, and I was then much censured for attributing blame for such occurrences. The opinions I then ventured to broach have been strenghtened by every year's experience; and I now fearlessly assert, that, by due attention to very simple principles of conduct, such deformities may generally be avoided. I shall now endeavour to explain those principles to you; and should I not make myself clearly understood, I request that you will not hesitate to ask for further explanation. I am quite ready to admit that it is not in our power to arrest the law of nature by which a cicatrized surface becomes smaller, and occupies less space, than the original wound; but it is in our power, in most cases, to direct and modify that which we cannot wholly prevent, and thus, at all events, to counteract its injurious effect. We cannot prevent the process of absorption which has been described, but we can prevent its taking place in a direction which may interfere with the healthy functions of the part. To take the upper extremity as an example, I will suppose a case where the whole integuments on the inner and front part of the arm and fore-arm have been destroyed. If such extremity be carefully kept extended on a splint, not only during the whole progress of healing, but long subsequent to the perfect cicatrization, you will find that the cicatrized surface will diminish in a circular direction, drawing the healthy integument together from side to side; but that no contraction will take place in the long axis, in which alone it can impede the due motions of the limb. This permanent extension should be persevered in during the day and night, until all changes have ceased, and the cicatrix has contracted to its smallest dimensions. Care, however, should be taken, during this time, to give passive motion to the different joints; by which the proper secretion of synovia will be kept up, and the eventual free use of the limb will be ensured." (P. 39.)

"It will be right, to offer a few observations on the structure and nature of the diseased and indurated cicatrices which follow the healing of burns. Whenever any unnatural contraction follows a burn, the cicatrix becomes more or less indurated and callous; sometimes to a most extraordinary extent, so as to merit the epithet of scirrhous. To what are we to attribute the more frequent occurrence of such indurated contraction after burns, than after any other description of ulcers? This is a question not easy to solve; but I believe it, in many cases, to depend on the constant state of irritation and chronic inflammation which is kept up by the continual stretching of the part affected, in the vain attempts made to extend the limb. That this may fairly be considered as one fruitful cause of such defects, may be inferred from the fact, that when a limb, under apparently similar circumstances, is kept extended during and after the healing, no such diseased cicatrix will result. Still, however, it must be admitted that, in some parts of the trunk, where no such contraction can operate, the cicatrices after burns form prominent ridges, and are morbidly hard; probably in consequence of the extensive destruction of the subcutaneous cellular tissue. This will at times amount to an increased morbid growth, to a considerable extent, which will have, when cut into, all the characters of a true scirrhus. Such a case occurred to me, some years since, in the person of the young female, of whose neck I now present you a drawing, exhibiting prominent pendulous tumors, one of which I removed, and approximated the healthy integuments, in hopes of obtaining union by first intention. Erysipelas supervened, and the surface granulated; and after it had healed, the same diseased growth returned nearly to the same extent. This is the only instance of the kind which I have met with, and it appeared to depend on some peculiarity of the individual, as the whole original cicatrix was freely removed. When such a horny web or cicatrix, as I have described, has contracted any joint, or the neck, it would appear, to a superficial observer, that the whole evil depended on the contracted integuments, by a simple division of which the limb would be instantly set at liberty. So deceptive is this appearance, that I have known, more than once, that surgeons have indulged this vain hope of affording relief, until a painful and ineffectual operation has convinced them of their error. In recent cases, occurring in any of the extremities, the contraction may be confined to the integuments, by dividing which the deformity may for a time be removed; but the same cause, continuing to operate, will produce the same effect, and the cicatrix will again contract after the wound is suffered to heal up. When the contraction has been of longer duration, the muscles acquire a new sphere of action, and afford an additional and powerful opposition to the free exercise of the limb. Lastly, in some cases we find that even the bony fabric becomes moulded and altered by the powerful constriction exerted on it by this gradual but certain process. In such cases it is hardly necessary to add, that the most severe operations cannot afford a prospect of even temporary alleviation. From having witnessed several such operations, and the repeated and ineffectual transverse division of such contracting bands, I was induced to adopt a different mode of proceeding in a case which fell under my care, at the Foundling Hospital, in the year 1813. Being aware of the inefficacy of the transverse incision, I removed the whole diseased cicatrix, and endeavoured to approximate the healthy integuments from the two sides of the arm, which was kept extended on a splint, not only during the healing of the wound, but for a considerable time after the new cicatrix had formed, until, indeed, all those changes which I have described had been fully accomplished. By such a practice, I conceived that the contraction, which I knew must follow so extensive a wound, would take place in a lateral direction, and not in the long axis of the limb: in a word, I hoped to be able to direct and modify that which it was not in my power to prevent. The success which attended this operation exceeded my most sanguine expectation. The boy's arm was perfectly restored, and remains straight to this day. The happy result which followed this operation, which was conducted on just principles, led me to consider how far those principles were applicable to the prevention of such accidents, by regulating the direction of the contractile process during and after the healing of large wounds in the neighbourhood of joints. It was quite obvious that the cases were precisely parallel, or rather, I should say, that the prevention was far easier to be effected than the cure." (P. 48.)

The following is an interesting case, and with its detail we shall take our leave of this pamphlet.

"It has been asked, at how distant a period from the receipt of the injury success may be obtained? This must, of course, depend upon the situation of the burn, and extent to which the muscles and bones are implicated in the mischief. I have, however, been truly astonished to find at how remote a period contractions of the fingers, hands, and arms, may be restored. A young lady, aged seventeen, applied to me for an affection of her spine: during my attendance, I observed that her right hand was much deformed, in consequence of very firm cicatrices which bound down her thumb and three fingers, so as to greatly interfere with the use of the hand. She had been burnt when quite an infant, and the defect had existed for nearly sixteen years, growing with her growth. From this history it was to be apprehended that the tendons and muscles would afford a serious obstacle to any improvement. I proposed, however, to make a trial with only one finger, and explained my views to the lady and her friends. She cheerfully acquiesced, as she was very desirous of being able to reach an octave on the piano-forte, being very fond of music. I succeeded so perfectly with the first finger that I proceeded with the others, and the thumb; and, eventually, restored the hand, and enabled my patient to attain the object of her ambition." (P. 53.)

---

*Improvements in the Microscope, by* Mr. Cornelius Varley *and* Mr. William Valentine; *with a Supplementary Letter from* R. H. Solly, Esq. *on certain Parts of Vegetable Structure.* [From the *Transactions of the Society of Arts*, vol. xlviii.]—8vo. pp. 97; three plates.

Messrs. Solly, Valentine, and Varley, are gentlemen all well and favorably known to the scientific world; and they have added much to the debt philosophers already owe them, by their successful labours to improve the microscope; an instrument which is daily becoming of more and more importance, revealing, as it does, wonders in the unknown world at home, as great in their minuteness as the worlds discovered by the telescope, beyond the boundaries of our solar system, are majestic in their grandeur.

In the pamphlet now before us, besides much useful and valuable practical information respecting the best methods of grinding and polishing lenses, there are detailed accounts of the structure and peculiar advantages of two microscopes; one by Mr. Varley, for the more convenient observation of living animalculæ, and another by Mr. Valentine, to facilitate the minute dissection of plants; both of which appear to us well calculated to fulfil the purposes for which they were designed. A particular description of these, however, could scarcely be rendered intelligible without the plates by which they are accompanied; and we, therefore, rather propose to make some extracts from the accounts which have been given of the observations that have been made with them, and more especially the verification of the discoveries of Schultz and Amici, of the visible motion of the sap in plants; a fact which, though long doubted and often denied, is now as demonstrable, and can be made as visible, as the motion of the blood in animals. The plant in which this phenomenon has been the most observed is one that was selected on account of its transparent integument; but the same or a similar motion may be seen in other plants besides the Chara. His Grace the Duke of Buckingham informs us, that Amici shewed it to him in the leaves of the common vine, and figures of the currents have been published, as seen in the stipules of the Ficus elastica and the Alisma plantago; and we learn that lately it has been also noticed in the Hydrocharis morsus ranæ, and the semipetaloid filaments of Tradescantia.

Mr. Varley has, we believe, examined this motion in the Chara more assiduously than any other person, and of it he gives the following account.

"In the month of May last, I was made acquainted with the chara by R. H. Solly, esq., who favored me with a portion, stating that the circulation could be seen in it with a good microscope. This I examined, and preserved it in a growing state, and soon saw the beautiful phenomenon of a circulation in various parts, and also in the new and very fine roots which it sent out from time to time. During the summer, Professor Burnett favored me with a plentiful supply, since which I have kept it growing in glass jars and phials; and now, in December, have got several portions of the plant, and a considerable stock of seedlings, many so far advanced as probably to need only suitable weather to begin shewing flowers and seeds." (P. 58.)

"In describing the chara, I will begin with such a portion as we are likely to find in its native place.

"These plants are found very numerous and entangled together, growing from the muddy bottom of water, two or three feet deep, where I obtained it. The stem is sometimes short between the knots, and sometimes as long as five inches. Nine similar and regular arms grow out on the top of, and with each length; from the midst of these grow out the second length, with its nine arms at top closed like fingers; and, as they open, a third length, and so on, keep growing out. From any of the knots, branches are liable to grow out: they are in every respect like the first stem; but whenever a knot sends out a branch, it also sends out very fine and perfectly transparent roots. The arms have four or five joints, from which the flowers and fruit grow.

"The stems are hollow tubes, which, in the most healthy state of the plant, are covered, for their whole length, with about eighteen smaller tubes. These outer tubes are frequently broken away, and, in taking a quantity from its native place, many lengths are found entirely deprived of their outer tubes, and uncovered, and the small arms either decaying or removed, leaving only the knots. These lengths, if left very quiet in water, will send out small branches from the knots; but in this case the first length is a naked tube, very transparent; and from the same knot perfectly transparent roots are sent out: they are small tubes. In this state the plant is an excellent microscopic object. The circulation to and fro is visible in the largest tube or stem; it is beautifully visible in the young naked branches, and, with a higher power, most perfectly visible in the fine roots; for they are quite as clear, colourless, and bright as glass tubes. Under favorable circumstances, the circulation may be seen in the large tubes with a quarter of an inch lens, but the tenth of an inch shews it quite well; for the roots and the small tubes the twentieth of an inch is better. All, except the roots, are studded or covered with minute cells, and are of a good green colour.

"The small tubes which cover the stem always wrap spirally round it. They sometimes are continuous tubes the whole length, from knot to knot, but sometimes they are jointed here and there, and some lengths are intercepted with numerous joints. These short lengths of small tube have a complete circulation up and down between each joint, and, when they separate from the main stem, they part at these joints, and remain whole, the circulation still going on. Though the cause of their parting appears to be owing to an injury done to some of the intermediate or lower lengths, in some cases these outer tubes become too long, by growing faster than the inner part or tube, and peel off on that account without being disjointed. I have also had some where the outer tubes appeared stationary, while the larger inner tube increased in length, and thrust itself out from the small tubes to bend like a bow, the small tubes appearing to be the strings that kept it bent. These accidents are very favorable for observing the different circulations.

"In its native place, the arms are mostly covered for their whole length with about nine small tubes, and these arms bear the flowers and fruit at each joint.

"I have many samples growing in-doors in which these arms are quite naked; they then bear no fruit. The outer tubes appear essential for that purpose; for to whatever joint the outer tubes extend, so far flowers and fruit are likely to appear. Around the knots and close under the arms there are eighteen pairs of small cells, two pairs under each arm; and occasionally there are short cells along the main stem, which grow out from between the surrounding tubes: they appear always to hang downwards, and are in some cases very numerous." (P. 58.)

"I have now described the plant, not beginning from the seed, but from such a portion as we are liable to find in its native place, and traced it through the bearing of seeds to their growth, into such a portion as I commenced with. I will now describe the circulation, beginning from the seed.

"As soon as the young plant has protruded from the seed, and grown a few times longer than its width, it shews all the parts, and circulation becomes visible in the stem. The portion between the roots may be considered as one whole cell, the fluid rising up one side, then across and down the other, then across and up again. If the fluid had been pure and limpid, it would have been difficult to discover it; but, fortunately, it is always crowded with various-sized particles, mostly spherical, some clustered together, and here and there a larger mass, so that the fluid appears sluggish, as if from being of a gummy nature. It is frequently like a dilution of the contents of the seed: the irregular lines, dots, or masses, within the lower portion, and the stem, shew the appearance of the matter which circulates. The stem, from root to top, may also be considered one whole cell, the fluid going spirally up one half and spirally down the other half, and across the top and bottom. I have fixed my eye on a particular mass, and followed it up and down several times, but with little change in its size and form. The inner portion of these tubes or stems is filled with fluid, without any visible density or motion." (P. 65.)

"The ends of all the roots are liable to be filled with a quantity of the thickening portion of the circulating fluid, the currents crossing over at the top of this portion; and when any of the largest masses touch here, they are so impeded that the smallest particles may be seen to overtake and pass them. The density of this mass is sufficient to make the end appear like a solid glass rod: the contrast at once proves the hollowness of the other portions, for they appear like glass tubes." (P. 67.)

"One remarkable circumstance in this plant is its extreme simplicity; for, excepting that the surface is studded with one layer of minute cells, and the knots are clusters of larger cells similar to the tubes, all the rest of the plant is composed of tubes differing in length, in every one of which is a separate and distinct circulation.

"The next remarkable circumstance is the universal spiral character, for the outer tubes wrap spirally around the inner ones. The circulation also makes spiral turns within the tubes, and the outside texture, of extremely minute cells, follows the same spiral direction.

"These spirals are all to the right, as a right threaded screw; but on the contrary, the tubes of the seed wrap spirally round to the left." (P. 67.)

In Mr. Solly's supplementary letter there is much that pleases us; and as it relates to several disputed points, and as the argument is very clearly and shortly stated, we feel that we shall oblige our readers by making some copious extracts. It relates, first, to the anatomy of the petal of the Anagallis; secondly, to the structure of spiral vessels, and their presence and absence in certain tribes of plants; and, lastly, to the physical cause of the motion of the sap.

"The veins in the petals of the Anagallis are composed entirely of spiral vessels. As many of our members are but little acquainted with vegetable anatomy, it may, perhaps, be proper here to explain what a spiral vessel really is. A spiral vessel consists of a very fine transparent membrane forming a cylindrical tube, closed at each end by a conical termination, and having one or more fine threads wound in a spiral direction round the inside of the tube: I think that these fine threads are themselves tubes, though that has been disputed, and they are so fine that it will require very nice observation to settle the point. The thread adheres so firmly to the tube, that, in unwinding or pulling it out, the tube is torn in a spiral direction between each coil of the thread, so that it has been doubted if there were a complete tube, independent of the spiral thread, or whether the membrane only connects the spiral coils of the thread; which really is much the same thing. By an inspection, it will be evident that, although the veins run in an uninterrupted line from the base of the leaf to very near the margin, yet that the spiral vessels of which they are composed are individually short compared with the length of the vein, each vein being composed of many spiral vessels; and where one spiral vessel terminates and another begins, the ends are contiguous and overlap each other, as if they were spliced together.

'Although the veins ramify, yet the individual spiral vessels of which they are composed never do so, but where the veins branch, the branches are formed either by one spiral vessel being spliced on to the side of another, or by two spiral vessels being spliced on to the end of a third on the opposite sides. * * *

"The corolla of the Angallis is entirely composed of spiral vessels and cellular tissue, enclosed in a membrane or cuticle."

"The oblong vesicles of cellular tissue are sometimes marked with spiral lines, in which case they seem to have some imperfect resemblance to spiral vessels. Ducts are said to be distinguished from spiral vessels principally by their being incapable of being unrolled; but ducts are often capable of being unrolled, though in that case they rather resemble ribands than threads. In the plates to a work by Kieser, entitled 'Mémoire sur l'Organisation des Plantes,' are some very beautiful and accurate representations of ducts, some of which are partly unrolled. Spiral vessels are probably sometimes converted into ducts by the threads partially adhering together. The terminations of ducts are similar to those of spiral vessels. I believe Mr. Valentine, by dissecting them out cleanly from the cellular tissue in which they are enveloped, was the first person who shewed clearly the terminations of spiral vessels and ducts, and the way in which their contiguous ends are joined to one another. I have got some preparations shewing this very clearly, which Mr. Valentine put up for me in spirits in the spring of 1830, according to the method described in the present volume of the Society's Transactions, and which still remain as perfect as they were when first put up. I believe the terminations of ducts and spiral vessels had been previously seen and described, though imperfectly, by other persons. Both ducts and spiral vessels may be beautifully seen and easily dissected out from boiled asparagus. It may perhaps tend to reconcile the contradictory opinions entertained by botanists, as to whether the sap rises through the cellular or the vascular tissue, to remark that, as ducts and spiral vessels are closed at both ends, they are in fact elongated cells. Spiral vessels are said to exist only in Phænogamous or flowering plants, and therefore, in the natural system of Botany, Phænogamous, or flowering plants, are called Vasculares; and Cryptogamous, or flowerless plants, are called Cellulares; but as Mr. Valentine found spiral vessels (though rather imperfect) in Filices, or ferns, this distinction is not quite correct. Perhaps the best arrangement would be to divide plants, as at present, into the two grand divisions of Phænogamous, or flowering plants; and Cryptogamous, or flowerless plants; and, leaving the subdivisions of Phænogamous, or flowering plants, the same as at present, to divide Cryptogamous plants into two subdivisions, Vasculares and Cellulares; particularly as ducts exist in a large class of Cryptogamous plants. I believe there is not at present any satisfactory account of the use of, or the functions performed by, spiral vessels; but perhaps by attending to the particular parts of plants in which they are found, some conjecture may be formed upon the subject. Spiral vessels are said to exist only in the medullary sheath, and in the parts which emanate from it in an ascending direction, as the leaves and the flowers (which are supposed to be only modified leaves), and not in any part that is formed in a downward direction, as the wood, the bark, and the root; the wood increasing only by successive additions to its exterior surface, and the bark by successive additions to its interior surface. I think the most accurate general expression for those parts of plants in which spiral vessels exist, would be, those parts which grow by an increase through the whole of their substance, or which elongate by the general extension of parts already formed. A root is said to increase in length only by the successive addition of new matter to its apex or point, and not by any extension of a part already formed: it is also said not to contain spiral vessels; but Mr. Valentine found spiral vessels in the root of the Agapanthus Umbellatus, or blue African lily, but only near its point; and I think it probable that some roots may occasionally grow to some extent by the extension of the parts near the end, which are recently formed, and not merely by the addition of new matter at the apex. If a bulb, (or a bulbous root, as it is erroneously called), the bulb of a hyacinth for instance, be cut open in the winter, or very early in the spring, it will be found to contain within it the whole plant, small, but perfectly formed, consisting of the leaves, the stalk, the flowers, with all their parts complete; so that the subsequent growth of the plant seems to be nothing but the evolution of parts previously formed.

"The hyacinth, particularly during the early period of its growth, will be found to consist principally of cellular tissue, and spiral vessels, or ringed vessels. A ringed vessel is a tube terminating in a cone at each end, in the same way that a spiral vessel does: the tube consists of a fine membrane, which is prevented from collapsing by equidistant internal rings. In different vessels these rings will be found to be at very different distances: they are sometimes so far apart that they appear to be loose in the cellular tissue; but I believe, if accurately examined, they will always be found to be enclosed in a tube. Ringed vessels have been supposed, perhaps erroneously, to be formed from spiral vessels, by the threads of the spiral vessels being broken by the stretching of the tube, and the successive coils of the threads collapsing into rings. Rings and spiral threads are often seen alternating with each other in the same vessel; they run so much into one another, that I think they may be considered merely as varieties of the same kind of vessel.

"Very good specimens of rings and spiral threads, alternating with each other in the same vessel, may be found in the stem of the Tradescantia Virginica, or Virginian spider-wort: ringed vessels may also be easily dissected out from the petiole, or leaf-stalk, of the common or culinary rhubarb, especially after it has been boiled. The leaves and stalks of some plants grow with astonishing rapidity: they will, on examination, be found to consist of a very small quantity of solid matter, not one tenth or perhaps sometimes not so much as one twentieth, the remainder being a watery fluid; and the solid matter will be found to consist principally of cellular tissue, and spiral or ringed vessels.

"Suppose a person had this problem to solve: what would be the best way of constructing a vegetable substance so that it shall be formed completely in a small space, and be capable of rapidly developing itself into a large plant? Such a substance could not be constructed better than of cellular tissue and spiral vessels, the cells being collapsed, and the spiral threads pressed close together; then, if by any process the cells were rapidly but gradually filled with fluid, a small stalk with minute buds might, in a short time, be expanded into a lofty stem, bearing large bunches of flowers, as the Agave Americana, or American aloe, which rises to the height of twenty feet and upwards in a very few days. I do not mean to assert that the whole of this growth consists merely of the expansion of the cells by their being filled with sap, and the consequent stretching or expansion of the spiral vessels; for the sap, at the same time that it expands the tissue, becomes partly assimilated to it, and thus increases the solid matter of the stalk. Though the stem is developed, it is not formed in a very short space of time. In its own native hot country the American aloe takes some years to form its flowering stem; and in this country, I believe, it seldom blossoms till it is forty or fifty years old. That there is a great flow of sap into the flowering stem of the American aloe at the time of its developement, is proved by the method employed for procuring the liquor called Pulque, which is the favorite beverage of the inhabitants of Mexico.* Just as the flowering stem begins to shoot up, it is cut off, and the centre of the plant is cut out so as to form a bowl, into which the sap which was intended for the developement of the flowering stem flows, and from which a considerable quantity of liquor is procured during all the time that the plant would have remained in flower. I think it is clear that the great influx of sap is the principal cause of the rapid developement of the flowering stem; but the cause of the influx of the sap is by no means clear. Perhaps the phenomena first observed by Dutrochet, and called by him Endosmose and Exosmose, (to which I shall have occasion again to refer,) may have something to do with it; though I do not see how they are capable of explaining it. It is said, that in a leaf there are two sets of vessels, one of which is on the upper and the other on the under side, and that they communicate with each other at or near the margin of the leaf, that the circulation is carried on by the sap ascending from the stem through the vessels on the upper side of the leaf, and returning by those on the under side. But it is clear that this cannot be the case in the corolla of the Anagallis, and in those flowers which are similarly constructed; for, on referring to the petal, it will be seen that there is only one set of veins, and that each vein is composed of only one line of vessels, except where the vessels overlap each other for a short distance at their junctions; the sap, therefore, cannot flow up through one set of vessels and down through another, but the circulation must be carred on in some other way, and I think probably by means of what has been called Endosmose and Exosmose, of which phenomena it will be necessary to give some explanation. If any two fluids, or if a solid and a fluid, having a chemical attraction for each other, are prevented from coming in contact by the intervention of any thin animal or vegetable membrane, they will pass through the membrane and unite with each other, although under ordinary circumstances the membrane would be impervious to one or both the fluids. There will always be two currents passing through the membrane in opposite directions, but the current will be the strongest from that fluid to which the membrane is the most pervious towards that to which it is the least pervious.

* There is a curious error in the Supplement to the Encyclopædia Britannica, where Pulque is said to be procured from a species of Cactus, or Opuntia.

"When a solid and a fluid are employed, there will also be two currents, provided the solid is soluble in the fluid; for in case it is not, there will only be a current in one direction from the fluid to the solid: where two solutions of the same substance (as sugar for instance) are employed, but differing in strength, there will be currents through the membrane in opposite directions, but the strongest current will be from the thin syrup to the thick, and by means of these two currents the strength of the two solutions will ultimately become equal. Let us try if we can apply these phenomena to explain the way in which the sap circulates in the petal of the Anagallis. The fluid with which the cells are filled is a mucilaginous juice; by the evaporation of the water this juice becomes thicker; but as the greatest evaporation will take place from that part of the petal which is nearest the outer edge, that part being the most exposed to the sun and air, the mucilaginous juice which is contained in the cells situated in that part of the petal will be the thickest: there will, therefore, be a current into those cells from the cells immediately adjoining them, in which the sap will be thinner; but there will also be a counter-current from the thick sap to the thin, tending to equalize the density of the sap in these two sets of cells; and as the sap in the second set of cells will thus become thicker than that in the cells immediately adjoining them on the side farthest from the outer edge of the petal, an interchange of sap will take place between these two sets of cells: thus there will be a constant circulation of the sap going on through all the cells whenever, from evaporation or any other cause, there shall be any difference in the density of the sap contained in any of the cells. There will be two currents traversing the whole of the petal, one from the base to the outer edge, and the other from the edge to the base: the current from the base will be the strongest, to supply the greater evaporation in that part of the petal which is most exposed to the sun and air. As the circulation can thus be carried on through the cellular tissue alone, it may be asked, what is the use of the spiral vessels? The spiral vessels being themselves cells, an interchange of currents will take place between them and the cells with which they are in contact, in the same way as it takes place between one cell and another; but the spiral vessels, being tubes, are in contact with many of the cells, between all of which and the spiral vessel a circulation may be going on; and, as there are lines of spiral vessels running all the way from the base of the petal to near its margin, the circulation can be carried on through the whole petal by means of these veins with much greater rapidity than it could by the cellular tissue alone: the lines of spiral vessels are also continued down the stalk, and thus serve to promote a similar circulation between the various parts of the flower and the footstalk on which it grows. The spiral vessels may be beautifully seen in the stamens of the Anagallis, running up the centre of the filament. Besides promoting the circulation, the veins probably tend to give stability and a determinate form to the petal, and serve to connect together the cells of the cellular tissue. I have endeavoured to point out what I conceive to be one of the causes which tend to promote the circulation of the sap, but I am well aware it cannot be the only cause; for it would act just as well upon dead vegetable matter (so long as it retained its organization) as it would upon living. If, therefore, it were the only cause, the sap would continue to circulate in a dead plant just the same as in a living one; and in the winter (at least when there was no frost) just as well as it would in the spring and summer, which we all know not to be the case; nor will it account for the great flow of sap during the time of the rapid developement of the flowering stem of the great American aloe, and which also takes place in other plants during the developement of their leaves and branches in the spring and early part of the summer.

"The laws of mechanical and chemical action to which living plants are subject, are so modified and controlled by those of vital action, that, until we are better acquainted with the laws of vital action, all attempts to explain the cause of the circulation of the sap and the growth of plants must necessarily be very imperfect. Though a knowledge of the laws of mechanical and chemical action will not alone be sufficient to explain the cause of the growth of plants, still it may give us considerable help towards attaining a knowledge of that cause. For instance, although Mr. Knight's very ingenious experiment (published in the Transactions of the Royal Society for the year 1806,) of causing beans to grown on a revolving wheel, did not explain the cause why the beans grew at all, that depending upon vital action; but the beans being in a state of growth, it clearly proved that the mechanical action of gravitation is the cause of the descent of the radicle or root, and of the ascent of the plumula or stem." (P. 74.)

We can only, in conclusion, again express the pleasure which a perusal of these papers has given us, and, in returning our thanks to our fellow labourers in this field of science, beseech them to continue their researches and render us still more their debtors.

---

*The Effects of Arts, Trades, and Professions, and of Civic States and Habits of Living, on Health and Longevity: with Suggestions for the Removal of many of the Agents which produce Disease and shorten the Duration of Life.* By C. TURNER THACKRAH, Esq. *Second Edition, greatly enlarged.*—8vo. pp. 233. Longman and Co., London; Baines and Newsome, Leeds.

BUT little more than a year has elapsed since the publication of the first edition of this very interesting work. In the present edition Mr. THACKRAH has added much important information to many of the chapters before published, and he has, besides, investigated the effects upon the health of artizans from 120 employments, to which he had not before adverted. The great claim that Mr. T. possesses to the attention of the philanthropist and physician is, that he writes from personal observation, and he has, with great industry, collected numerous important facts, which cannot but be worthy the attention of every man who feels for, and who would gladly alleviate the distresses of those to whom all are so deeply indebted, namely, the "operative" classes of society. To the great capitalist and master-manufacturers, who have in their employment numerous workmen, Mr. Thackrah's comments will be found especially valuable. It is their interest, and we trust it is generally their inclination, to do all in their power to secure the health and happiness of their labourers. The latter, if left entirely without control, are usually reckless of their own health: provided they earn sufficient for the present moment, they rarely think of endeavouring to alleviate any of the inconveniences or even positive injuries they suffer from the effects of their respective occupations; and too often, by habits of irregularity and intemperance, they reduce the natural powers of their constitutions and render themselves easy and early victims to trials, incidental to their trades, which would make little or no impression upon men in perfect health. That this is really the fact, must be too obvious to require any formal proof; and if such were necessary, it is amply afforded by Mr. Thackrah, who finds frequent occasion to comment upon the readiness with which the intemperate workmen suffer in comparison with the sober. A kind and skilful master may do much for the men, which they will not do for themselves. In the first place, as Mr. Thackrah shews, in many instances the injurious effects of different arts and trades may be diminished by simple expedients, and no doubt the moral conduct of the "operatives" may be greatly improved by holding out encouragement, and granting little indulgencies, to men of sober and steady habits, which would be withheld from those whose conduct was less praiseworthy. The independent spirit which characterizes the working classes of society would render nugatory any harsh attempt even to make them consult their own health and happiness; but they would fully appreciate, and feel grateful for the kind concern evinced by their masters in their welfare; and, with but a moderate dexterity in enforcing them, they might be induced to submit to regulations which were clearly for their benefit, and for their benefit alone. It is well said by Mr. Thackrah, that "the vice of the operative reflects upon the master." "A master can, a master ought, to interfere;" but he must so interpose his interference as to convince his workmen that his object is to secure their health and comfort by leading them from habits which are destructive to both. A few may be altogether irreclaimable; but, in the majority, sufficient good sense and good conduct will be found to second the philanthropic efforts of the master.

In order that these efforts may be skilfully exerted either as regards the diminution or prevention of the evils to which the men are exposed in their respective employments, or as regards their moral management, Mr. Thackrah's work must be consulted. We believe it is the only one in the English language upon the subject; and we cannot sufficiently applaud the industry and zeal which has enabled Mr. T. to collect so much important and valuable information concerning it. In our number for June 1831 we gave a brief outline of the contents of the first edition of this work. We shall now confine ourselves to the leading facts contained in the additional pages which so materially increase the value and utility of this second edition.

"The *Brass-founders* suffer from the inhalation of the volatilized metal. In the founding of *yellow* brass in particular, the evolution of oxide of zinc is very great. It immediately affects respiration: it less directly affects the digestive organs. The men suffer from difficulty of breathing, cough, pain at the stomach, and sometimes morning vomiting. The brass melters of Birmingham state their liability also to an intermittent fever, which they term the brass-ague, and which attacks them from once a month to once a year, and leaves them in a state of great debility. As a preventive, they are in the habit of taking emetics. They are often intemperate. In Leeds we did not find one brass-founder more than forty years of age; though we have since been informed that there are two brass-founders in the neighbourhood, of the ages of sixty and seventy, who have continued at the employ from boyhood. The *Turners, Filers,* and *Dressers of Brass,* if confined to this metal, do not seem to be more unhealthy than the generality of our townsmen. We observe among the filers the hair of the head changed to green. This I suppose to result from the oil of the hair combining with the copper in the brass particles." (P. 101.)

*Bronzers,* it appears, suffer no annoyance from the frequent immersion of their hands in mineral acids. *Copperplate printers* use considerable muscular exertion. In blue printing, Mr. T. states that a composition of white lead, Prussian blue, and turpentine, is employed, instead of common ink; and the hands of the workmen are often daubed with the paint from morning to night: yet no injury appears to result, no case of colic or palsy from the poison of lead has been found among these workmen. Whence, Mr. Thackrah asks, their exemption? An intelligent master thinks the mixing of the mineral with boiled rather than cold linseed oil may prevent its poisonous effects.

"But this is scarcely probable. Does the prussiate of potass exert any antidotal effect on the oxide of lead? We gave a compound similar to that of the printer's blue to a dog, with the same fatal result as if a like quantity of oxyde of lead had been given in any other form. On the whole, I am inclined to attribute the exemption from palsy to the comparative infrequency of the process; the printing with blue ink being much more rare than the printing with black. Engravers and copper-plate printers, though not remarkable either for temperance or excess, present few examples of old age." (P. 44.)

*Weavers of Coverlets* are exposed to considerable dust from the chalk with which the web is imbued. Though they do not complain of direct injury, or even of much annoyance, they are not only pale and thin, like weavers in general, but are subject to cough and difficulty of breathing. They do not commonly live beyond the age of fifty. The labour is much greater than ordinary weaving, on account of the larger size of the shuttles. The pressure on the pit of the stomach frequently produces painful affections of this organ.

"Of the *Dressers of Japanned Goods*, the few who are employed in *turning* inhale much fine dust. Pallor, sickness, impaired appetite, difficulty in breathing, cough, and expectoration, are the results. Few men, if any, bear the employ constantly for many years." (P. 85.)

"*Engravers* fix the trunks and limbs more than almost any other operatives. The head is brought forward, and the eye intensely and long occupied with objects generally so small as to require a strong artificial lens. In one part of the process, the engraver is subjected to the annoyance of nitrous fumes, but this is only occasional. The posture and confinement affect the head, but more frequently, and more considerably, the organs of digestion. Sometimes the appetite is reduced, almost always the action of the bowels is greatly impaired. Organic diseases, however, of the abdominal viscera are by no means so frequent as in many other sedentary occupations, tailors and shoemakers for instance. This I attribute to the less general intemperance of engravers. The employment affects vision. Young men, for a short time after removing the lens, are unable to judge accurately of the relative size of objects, even at a foot's distance; and the eyes of old engravers are considerably impaired, both as optical and vital instruments." (P. 43.)

Mr. Thackrah briefly mentions the case of a Mr. B., now about the age of sixty, who was closely employed in engraving for thirty years His right eye, that which he applied with a convex lens to his art, is considerably more prominent than his left; and he is consequently obliged to close it when he looks at distant objects. Though not of late years engaged in engraving, he cannot accurately estimate the distance and relative position of near objects. In playing at backgammon, for instance, he frequently takes up a wrong man. In weak light the left eye is better than the right. "Cases of this kind illustrate some points of function and disease."

*Farriers* do not, Mr. T. believes, suffer from handling the legs of horses affected with the grease; but he says that they are liable to absorb the poison of glanders, and several cases are recorded of human death from this horrible disease.* A man, with a scratch or wound of the finger, dresses a glandered horse; sometime after, the finger becomes painful; irritation in the absorbents succeeds; the constitution becomes affected; a disgusting suppuration takes place in the nostrils and throat; the air-tube, and

* Vide our Journal for December 1830.—Editors.

probably the lungs also, are affected, and death soon removes the sufferer. The importance of plastering any wound of the hands is quite obvious. When this precaution has been neglected, perhaps washing immediately after touching the diseased animal, and then applying a drop of nitric acid to the sore on the hand, would diminish the risk of absorption.

"*Flock-dressers*, chiefly women, are exposed to considerable dust: they would be to more, were not the first part of the process performed by a covered machine. The subsequent sieving and examining of flocks produces great dust, and decidedly injures both respiration and digestion. In proportion to the degree and continuance of this deleterious agent is the head affected, the appetite reduced, respiration impeded, cough, and finally bronchial or tubercular consumption induced. In summer, when the windows are open, the women suffer much less than in winter, when they are closed. Surely, for such dusty processes, either machinery should be more employed, or at least free currents of warm air be admitted to the operatives." (P. 66.)

*Water-gilders*, who coat silver or other metal with an amalgam of gold and quicksilver, are exposed to the same poison as the silverers of mirrors.*

"They diminish its effects, however, when employed on small work, by interposing glass between the mouth and the materials; and, when engaged on larger articles, by affixing to the mouth and nose a kind of proboscis, which, hanging down, opens at a distance from the source of the mercurial fumes. Notwithstanding these contrivances, and every attention paid to ventilation, the art cannot be closely pursued without the induction of serious disorder. Depression of spirits, or 'nervousness,' is succeeded by trembling, sickness, depraved taste, fetid breath, and, finally, salivation. Palsy also is frequent; but this, as well as the other maladies, is in most cases removed by rest and fresh air. Repeated attacks, however, destroy vigour of constitution, and shorten life. Men past middle age suffer so much more than others, that scarcely any are found at the employ. Water-gilders generally work but four days a week, and for about nine hours each day.

"Personal cleanliness and change of dress considerably diminish the bane of this and the preceding employment. Ventilation also, and the management of currents of air through the workshops, should be regarded as much as possible. When tremors appear, rest, fresh air, and aperients should be promptly employed; and for salivation, I have found opium the most efficacious and speedy remedy." (P. 113.)

*Gilt-button makers*, in the casting department, are subjected to great heat, and to rather severe effects from the fumes of zinc. The men have the appearance of ill health, and forty-five Mr.

* For an interesting article upon this subject, by Mr. MITCHELL, see our Journal for November 1831.—EDITORS.

Thackrah believes to be about their average duration of life. In gilding, the temperature of the room is 110 to 120 degrees: but the principal evil is the mercurial vapour.

"*Preparers or Dressers of Hair*, men, women, or boys, are in an atmosphere of dust and stench, especially when employed on the foreign article. The winnower suffers most. The complexion is soon rendered pale, the appetite reduced, the head affected with pain, respiration impeded, cough and expectoration established, the body emaciated. I scarcely need add, that life is sacrificed to a continuance of the employ. In most baneful arts and occupations, the wages are high; but here we find, with surprise, that the winnower does not earn more than 4s. 6d. or 5s. a week. For what a pittance is health broken and life destroyed! But why should the winnowing be effected by hand at all? why not employ machinery to turn the fan? or why not collect the dust in a box, and carry it off through a wooden chimney, by the current from the fan? Few persons, indeed, are employed in the dressing of hair, and fewer are acquainted with their situation and suffering: this may palliate, but cannot excuse, the neglect." (P. 69.)

The *Manufacturers of White Lead* are subjected to its poison both by the lungs and skin. Mr. Thackrah asks, will any chemical process avail to prevent the poisonous effects of this mineral? and can any substitute be found for its use in our arts and manufactures? To prevent the injurious effects of lead, the greatest cleanliness should be observed by the workmen. The success of this simple measure at one manufactory,* warrants the belief, that more than half the diseases of lead-preparers would be prevented by washing and brushing the hands and skin whenever they leave work, cleaning the mouth, changing the dress, and the regular use of the bath.

"A linen dress is also recommended, as excluding from the skin much of the dust which would enter through woollen. The rooms in which the processes are carried on ought, of course, to be spacious and well ventilated, and there should always be a strong draught through the furnace. A subsidiary chimney, anterior to the ordinary one, is mentioned by Dr. Christison as particularly efficient in carrying off the exhalations from the rakings. Men should never be allowed to take their meals in the workshops. Fatty aliments are recommended as a preservative from the poison of lead." (P. 106.)

As a proof of the fallacy of the popular opinion among workmen, that hard labour requires hard drinking to support their strength, we may just observe a fact mentioned under the head of *Marble Workers*. These men have great but varied exertion, and they profess to believe quantities of ale necessary to the performance of their work; though one of the most laborious, as well as most

* Alcock on the Education of the General Practitioner.

healthy, of their body in Leeds was known to take nothing stronger than milk.

Notwithstanding the disgusting nature of their occupation, the *Nightmen* of London are generally healthy. Of eighteen examined by Mr. Thackrah's assistant, only two had even slight disorder; appetite, they declare, is increased by the effluvium. In proof of the innocuous nature of putrid exhalations, Mr. T. quotes an interesting article, which we published in our Journal for March 1829, which shews that persons employed in occupations in which they are surrounded by, and almost live in, a putrid atmosphere, enjoy remarkable health, and are exempt from many diseases to which others are liable who enjoy a purer air.

"*Shoddy-grinders* (a provincial term,) are persons employed in certain districts of the West Riding of Yorkshire, as that of Batley and Dewsbury, in picking and tearing woollen rags, and afterwards manufacturing them, with the addition of new wool or worsted, into yarn. This is taken from the mill, and woven, at the houses of the workmen, into a coarse cloth or drugget. The only part of the manufacture which differs materially from the ordinary woollen, is the sorting and breaking up of the rags. Much dust is produced, particularly by the tearing machines, or 'devils.' For the removal of this there is, however, a very valuable, though but partially effective provision. A large box at one end communicates with the machine, and at the other with the open air, by means of a wooden chimney, which traverses the roof. The dust is driven, by a strong current, from the machine into the box, where the heavier and valuable parts subside, and the lighter pass through the chimney out of the building. Sometimes, however, a direction of the wind prevents the success of this contrivance, and the room is so clouded that persons cannot see each other at the distance of a few yards. Could not the dust be promptly diminished by a revolving end affixed to the wooden tube, where it opens outside the building; an apparatus similar to that frequently adopted for common chimneys?

"Girls and women are generally employed in sorting the rags; boys and men attend the machine. The boys at first are affected by the dust, but after a time they endure it without complaint. Men suffer more, and those who commence the employ after the adult age are often obliged to abandon it: indeed, very few persons remain for several years at the machine. My intelligent friend and quondam pupil, Mr. Breary, of Dewsbury, has favored me with a list of seventeen shoddy makers, whom he examined or professionally attended; and of these one only had been at the manufacture since its establishment in the district, thirteen or fourteen years ago; none of the others more than six years, and the majority not more than two. Mr. Breary remarks, that persons commencing or returning to the employ are so generally attacked with headach, sickness, dryness of the fauces, and difficulty of breathing, that the complaint is known in the district by the name of the 'shoddy fever.' This disorder subsides in six or eight hours; but cough and expectoration of dirty mucus, chiefly in the morning, generally remain, and indeed are almost universal, in a greater or less degree, among those who long and steadily attend to the machines. Difficulty of breathing, however, and tightness in the chest, do not appear to precede these symptoms for so long a period as in some other dusty occupations. Men, on returning to work after some days' absence, are always distressed for a time by the dust. Mr. B. mentions that the oldest man, aged thirty-nine, in his list, when respiration is more than commonly oppressed, finds prompt relief from citrate of ammonia in the act of effervescence, and from taking fatty aliments." (P. 67.)

*Soapboilers*, exposed to exhalations from the oil and alkali, are healthy, and even ruddy. During the plague in London, this employ was said to be remarkably exempt from the disease.

*Soldiers* are kept sober by strict discipline, and are generally a healthy body of men.

"Formerly a certain sum was paid to the soldier, from which he provided himself with food; but he so often spent in liquor what he ought to have spent in meat, that it became necessary for an officer to inspect his meals. A story current among soldiers illustrates the preceding statement. An officer going round the dinner-table of the men, saw one without meat before him. 'Donald, where is your meat?' 'O, here it is, sir,' shewing a vessel of slop, containing a mass of something like tripe. Day after day the same appearance was presented, till the officer, having some suspicion, demanded the exposure of the meat. 'O, it is tripe, sir,' said Donald. 'What, do you eat tripe every day? I must see it.' On striking a fork into the mass, the officer continued, 'Well, Donald, I never before saw tripe with buttons on it.' In fact, the meat proved to be a slice of leather smallclothes." (P. 19, *note*.)

If our limits permitted, we could extract many other interesting portions from the new matter contained in the second edition of Mr. Thackrah's work. It is proper we should state, that we have not followed the arrangement he has adopted in his account of the effects of the different arts and occupations on the health of the operative classes: we have selected some of the new articles as they appear in the index. We earnestly hope that the important investigation Mr. Thackrah has so ably conducted will be followed up by other observers, whose situations afford them opportunities of becoming acquainted with the prejudicial influences of different trades, and the best and easiest modes of diminishing, or altogether removing them. That this work will attract the attention of the masters of our manufacturing establishments, we cannot doubt: it would be a libel upon them to suppose for a moment that they can be indifferent to the topics discussed in it; but we beg to suggest, that it would be a great act of humanity to recommend it to the workmen themselves, who may not become acquainted with it unless it is particularly pointed out to them. To them the perusal of it must be very advantageous; for they will be taught how much their health and happiness depends upon their own conduct, and how very easily they may diminish many evils incidental to their various occupations, if, indeed, they cannot altogether avoid them.

